# Post-operative Unilateral Visual Loss and Ophthalmoplegia following Cervical Spine Surgery in Prone Position: A Case Report

**DOI:** 10.31729/jnma.8582

**Published:** 2024-05-31

**Authors:** Samaj Gautam, Suzit Bhusal, Ashlesha Chaudhary, Reshika Shrestha, Badri Rijal, Prakash Darjee, Surya Bajra Lama

**Affiliations:** 1 Department of Orthopedics and Trauma Surgery, National Trauma Center, National Academy of Medical Sciences, Kathmandu, Nepal; 2 Research and Development Unit, National Trauma Center, National Academy of Medical Sciences, Kathmandu, Nepal; 3 Maharajgunj Medical Campus, Institute of Medicine, Kathmandu, Nepal; 4 Department of Orthopedics and Trauma Surgery, Bharatpur Hospital, Chitwan, Nepal

**Keywords:** *case reports*, *central retinal artery occlusion*, *cervical spine surgery*, *prone position*, *unilateral visual loss*

## Abstract

Visual loss following a spine surgery in a prone position is a disastrous and irreversible complication. Moreover, the recommended treatment for such visual loss is lacking and the outcome is not so satisfactory. A 38-year-old gentleman developed profound right sided visual loss after an uneventful cervical spine surgery in a prone position that lasted approximately two and half hours. Immediate ophthalmic consultation was done and the case was diagnosed as right-sided central retinal artery occlusion. Despite the initiation of vasodilatation, anticoagulation, and adequate fluid infusion, satisfactory improvement was not achieved. Extensive review of pertinent literature highlighted limited efficacy of treatments for postoperative visual loss after prone spinal surgery, further emphasizing the importance of preventive measures as the cornerstone in such procedures.

## INTRODUCTION

Visual loss and ophthalmoplegia are very rare and devastating complications after spine surgery in prone position.^1,2^ Postoperative visual loss (POVL) has a reported incidence of 0.01 to 0.1% after prone spinal surgery.^3^ Causes include ischaemic optic neuropathy (ION), central retinal artery or vein occlusion (CRAO/CRVO) or occipital stroke.^3,4^ Factors such as patient positioning, hypotension, intraoperative blood loss, prolonged surgery time, anemia, diabetes, smoking, and vascular disease may be the cause of POVL.^2,4,5^ Early diagnosis and intervention could reverse visual loss. Here we report a 38-year-old chronic alcoholic with cervical spine injury who developed right-sided CRAO post-surgery, and was managed with high-dose dexamethasone, fluid resuscitation and anticoagulant therapy.

## CASE REPORT

A 38-year-old gentleman, chronic alcoholic, presented with a history of fall injury. He sustained injury to the neck following which he was unable to move right upper limb. There was no history of loss of consciousness, seizure or ear nose throat bleeding at the time of injury. After imaging with X-rays and magnetic resonance imaging (MRI), he was diagnosed as subluxation of cervical fifth (C5) over cervical sixth (C6) vertebrae with spinal cord contusion over C4-6 area. He underwent posterior instrumentation, decompression and fusion of C4-6 vertebrae. Surgery was done in a prone position with his head resting on horseshoe head rest. The duration of surgery was two hours and forty minutes. Estimated blood loss was around 300 ml. During the surgery, the mean arterial pressure was maintained at around 90 mm Hg and there were no events like cardiac arrest, arrhythmias, hypotension or decreased O_2_ saturation. He was transfused 2500 ml of normal saline during the surgery. Post-operative blood investigations including hematocrit were normal.

On the first postoperative day, he complained of loss of vision on the right side and redness of the right eye. Immediate ophthalmic consultation was done. His vision test revealed only normal light perception on the right side, which indicates that the patient could perceive light but could not distinguish shapes or objects suggesting severe impairment in his visual acuity. On the left side, his visual acuity measured 6/6. On external ocular examination, there was mild palpebral swelling and conjunctival congestion on his right side. There was ptosis, extraocular movements were limited in all the gazes on the right side, and the Extraocular Muscle (EOM) grading was -4. Intraocular pressure was measured using tonometry and was found to be normal. Mild corneal edema was apparent on slit-lamp examination. There was no proptosis. His right pupil was moderately dilated with Relative Afferent Pupillary Defect (RAPD). Fundoscopic examination of the right eye revealed retinal edema, a central cherry-red spot at the macula and attenuated arteries which were suggestive of CRAO. The anterior and posterior segmental examination of the left side revealed no abnormality.

**Figure 1:**
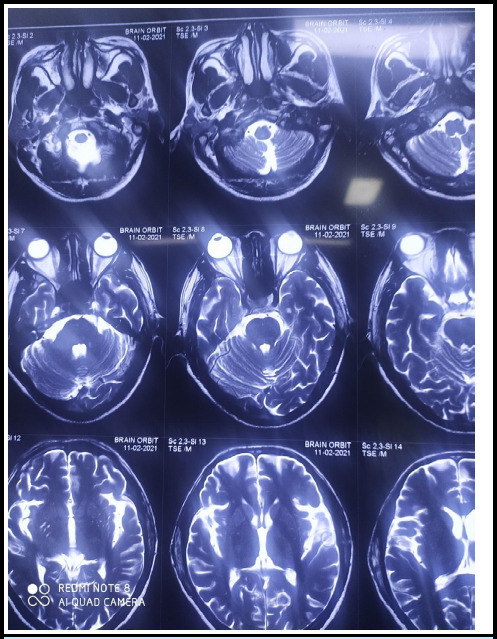
MRI of the brain showed no acute infarction on the brain. MRI of both eyes showed increased bulk and altered signal intensity in all rectus muscles of the right eye in comparison to the left showing T2/STIR high signal intensity.

Upon diagnosis of CRAO of the right eye, immediate administration of single high-dose methylprednisolone 1 gram was given to reduce inflammation and edema within the retinal tissues. Adequate fluid resuscitation with colloids was provided, and oxygen saturation levels were carefully maintained. Clopidogrel 75 mg was also promptly commenced to prevent further thromboembolic events and improve blood flow to the affected area and was further continued for 1 month. Continuous monitoring of vital signs, including blood pressure and oxygen saturation, was conducted throughout the management process. Daily visual charting was diligently conducted to monitor changes in visual acuity. Complaints related to the eye such as pain and discomfort were promptly addressed and managed accordingly with analgesics and ocular lubricants. Post-operatively at three months of follow-up, his ocular mobility has improved but no improvement in vision. He has shown improved physical mobility and is now capable of walking without assistance. According to the ASIA classification, his neurological status is categorized as ASIA E, indicating normal function.

## DISCUSSION

This case report describes a 38-year-old chronic alcoholic gentleman who developed profound rightsided visual loss after undergoing an uneventful cervical spine surgery in a prone position. Immediate ophthalmic consultation revealed a diagnosis of right-sided CRAO. Despite initiating prompt treatments such as high-dose dexamethasone, fluid resuscitation, and anticoagulant therapy, satisfactory improvement was not achieved. The patient's ocular mobility improved after three months of follow-up, but there was no improvement in vision. Vision loss following non-ocular surgeries is a rare occurrence, with limited documentation in the literature, particularly when attributed to CRAO. Moreover, cases presenting with MRI abnormalities in extraocular muscles are even more infrequent.

Postoperative vision loss after spine surgery is a rare but very devastating complication that affects the quality of patient life. Causes of POVL includes ischemic optic neuropathy (ION), central, CRAO, CRVO, occipital stroke.^2,4^ According to American Society of Anesthesiologists (ASA) the most common cause for POVL is ION (89%).^6^ According to the literature given by Postoperative Visual loss study group of the ASA, the risk factors for POVL include prolong operation time, excessive blood loss, male sex, obesity, administration of lower percentage of fluids and the use of Wilson frame.^7^

The most common cause for CRAO is malpositioning of patient head resulting in external eye compression which increases the intraocular pressure to such level that decreases or rather stops the flow in central retinal artery.^8^ The precipitating factors are hypotension, anemia, diabetes, improper patient head positioning, excessive blood loss, blood coagulation disorder. Facial abnormality, osteogenesis imperfecta, exophthalmos increases the vulnerability for external compression.^9^ The diagnosis of central retinal artery occlusion in this patient aligns with the literature, which suggests that malpositioning during surgery can lead to external compression of the eye, compromising ocular blood flow and resulting in ischemic events. Only a few cases of CRAO after spine surgeries have been reported in literature. Out of 27930 cases, Little et al^2^ found CRAO in only three cases. Retrospective review done by Stevens Et al showed 0.2% incidence of visual loss (ION, CRVO and Occipital infarct) but none showed CRAO.^1^

Few preventive and therapeutic measures for POVL have been discussed in the literature. The horseshoe-type headrest is not recommended for complex cervical and upper dorsal surgeries as it leads to increased direct pressure to the eye and it has been supported by study done by Roth et al.^7,10^ The study done by Asok et al^11^ highlighted the use of Mayfield clamp during surgeries to reduce the external eye compression. The management approach, including high-dose dexamethasone and anticoagulant therapy in our case, is supported by the goal of reducing the inflammation and promoting vascular perfusion to improve visual outcomes.^12,13^

It is very important that surgeons should think of perioperative vision loss as a dreaded complication and should be clearly discussed with the patient before the surgery. It should be highlighted in the consenting process. Both the surgeon and anesthesiologist should be aware of eye positioning and should be watchful of the perioperative blood pressure, duration of surgery and any other risk factors. Proper preoperative assessment minimizing the risk factors, correction of anemia, proper positioning, and proper intraoperative management and optimizing the blood pressure should be the priority. During surgery, the eye should be checked at a regular interval. The primary strength of our report is that the discussion of potential risk factors and preventive measures evident from the case provides useful information for clinicians involved in spine surgery. However, limitations include the absence of long-term follow-up data on the patient's visual outcome and the lack of comparative analysis with similar cases.

Ophthalmoplegia and CRAO are rare but grave complications after spine surgery. Postoperatively signs of periorbital swelling, conjunctival congestion, and ocular immobility in the eye could be the evidence of external eye compression intraoperatively. Preoperative identification of patients with high risk factors, patient counseling, correct positioning, intraoperative vigilant observation, maintaining hemodynamic stability, and proper postoperative follow-up followed by prompt treatment is highly advised. The limited knowledge about the causes of postoperative vision loss after spine surgery and its unsatisfactory outcome, till date emphasizes on further detailed research on this topic to shed clarity on the management strategies.
